# Association between thrombocytopenia and development of acute respiratory distress syndrome

**DOI:** 10.1186/s12931-025-03444-x

**Published:** 2026-01-13

**Authors:** Elpida Charalampaki, Konstantinos Gkirgkiris, David R. Price, Eleni Papoutsi, Georgia M. Minatsi, Georgia Dimopoulou, Stylianos E. Orfanos, Ioanna Dimopoulou, Anastasia Kotanidou, Ilias I. Siempos

**Affiliations:** 1 First Department of Critical Care Medicine and Pulmonary Services, Evangelismos Hospital, National and Kapodistrian University of Athens Medical School, 45-47 Ipsilantou Street, Athens, 10676 Greece; 2 Department of Medicine, New York-Presbyterian Hospital-Weill Cornell Medical Center, Weill Cornell Medicine, New York, NY USA; 3 Department of Medicine, Division of Pulmonary and Critical Care Medicine, Weill Cornell Medicine, New York, NY USA; 4 National and Kapodistrian University of Athens Medical School, Athens, Greece

**Keywords:** Acute hypoxemic respiratory failure, Acute lung injury, Platelets, Intensive care unit, Critical illness

## Abstract

**Background:**

Given that platelets support the integrity of the alveolar-capillary membrane, it is conceivable that thrombocytopenia may be associated with development of acute respiratory distress syndrome (ARDS). Yet, clinical studies confirming such an association are limited. We endeavoured to examine whether thrombocytopenia is independently associated with development of ARDS in critically ill patients.

**Methods:**

First, we performed a systematic review and meta-analysis of observational studies reporting the number of patients at risk for ARDS with versus without thrombocytopenia who eventually developed ARDS. Next, we performed a secondary analysis using individual patient-level data from three large randomized controlled trials to estimate whether thrombocytopenia (defined as < 100,000 platelets/µL) was independently associated with development of ARDS.

**Results:**

In the meta-analysis, four observational studies (five cohorts) involving 3666 critically ill patients were included. Patients with versus without thrombocytopenia were more likely to develop ARDS [46.3% versus 33.2%; relative risk 1.41, 95% confidence intervals (CI) 1.23–1.63; *p* < 0.001]. In the secondary analysis, data from 2927 critically ill patients were analyzed. After adjustment for confounders, including severity of illness, thrombocytopenia was not independently associated with development of ARDS (odds ratio 1.57, 95% CI 0.95–2.60; *p* = 0.080). The association between platelet count (as a continuous variable) and development of ARDS was non-linear and appeared U-shaped.

**Conclusions:**

Thrombocytopenia may not be independently associated with development of ARDS after adjustment for important confounders, such as severity of illness. Thrombocytopenia may serve as a marker of severe illness and its association with ARDS may not represent a mechanistic relationship.

**Supplementary Information:**

The online version contains supplementary material available at 10.1186/s12931-025-03444-x.

## Introduction

Acute respiratory distress syndrome (ARDS), a clinical entity with non-negligible attributable mortality [1], is characterized by leakage of protein-rich fluid due to disruption in the integrity of the alveolar-capillary membrane [2, 3]. The integrity of the alveolar-capillary membrane is supported, under normal conditions, by platelets [4]. Platelets may preserve the integrity of the endothelial barrier through mechanisms, such as physical obstruction of gaps in the vascular lining [5], release of molecules that help maintain the endothelial integrity [6], and promotion of growth of the endothelial cells [7]. The abovementioned mechanisms may be compromised in case of thrombocytopenia. Indeed, thrombocytopenia has been associated with increased permeability of the pulmonary capillaries [8] as well as thinning and fenestration of the endothelial cells [9] in animal studies of experimental acute lung injury. Yet, clinical studies confirming the association between thrombocytopenia and development of ARDS are limited.

On the other hand, thrombocytopenia is often the result of hematologic malignancy, which may also cause neutropenia. Neutropenia is associated with development of ARDS, as previously shown by others [10, 11] and our research team [12, 13]. Therefore, hematologic malignancy (especially if it results in neutropenia) might act as a confounder of the association between thrombocytopenia and development of ARDS. The latter association may also be confounded by severity of illness; that is, thrombocytopenia may merely serve as a marker of systemic illness and its association with ARDS (if any) may not represent a direct mechanistic relationship.

Keeping the above considerations into mind, we endeavoured to examine whether thrombocytopenia is associated with development of ARDS in critically ill patients, even after adjustment for confounders, such as hematologic malignancy and severity of illness.

## Methods

The present study consisted of two components: namely, a “meta-analysis” component and a “secondary analysis” component. The former is a study-level meta-analysis of observational studies. The latter is a secondary analysis of individual patient-level data from subjects enrolled in three recent randomized controlled trials.

### Meta-analysis

We reported the systematic review and meta-analysis in accordance with the Preferred Reporting Items for Systematic Reviews and Meta-Analyses (PRISMA) statement. We prospectively registered the protocol with PROSPERO (CRD42024570747) [14].

### Study selection, data extraction and outcome in the meta-analysis

Two authors (EC and KG) independently searched MEDLINE (via PubMed) and EMBASE using Boolean logic to create the following key phrase: thrombocytopenia AND (ARDS OR “acute respiratory distress syndrome” OR “acute respiratory failure”). We retrieved eligible articles published in any language up to July 6th, 2024. Given that “thrombocytopenia” might be considered as a too precise term, we repeated our search using the key phrase: platelet AND (ARDS OR “acute respiratory distress syndrome” OR “acute respiratory failure”). We considered as eligible observational studies, which enrolled adult critically ill patients with at least one risk factor for ARDS and presented data on the number of patients with versus without thrombocytopenia who eventually developed ARDS. We excluded interventional studies, case reports, case series, in vitro and animal studies, commentaries, editorials and reviews. We also excluded studies enrolling exclusively outpatients and studies enrolling patients with thrombocytopenia associated with extracorporeal membrane oxygenation, tropical or specific infections, disseminated intravascular coagulation and usage of heparin.

Two authors (EC and KG) independently extracted from each eligible publication the following data: first author or acronym, country, year of publication, study design, number of included patients, definition of thrombocytopenia, definition of ARDS, risk factors for ARDS, potential confounders on the association between thrombocytopenia and development of ARDS, and number of patients who developed ARDS among those with versus without thrombocytopenia. For the meta-analysis, thrombocytopenia and ARDS were defined according to the authors of each eligible study. Any disagreements regarding study selection and data extraction were discussed with a third author (IIS) and were resolved through consensus.

The outcome of interest in the meta-analysis was development of ARDS.

### Risk of bias assessment

Two authors (EC and KG) independently assessed the risk of bias in each eligible study by using the Newcastle-Ottawa Quality Assessment Scale [15]; a tool with broad application and established use in meta-analyses of observational studies [16, 17]. This Scale consists of three broad domains; namely, the “Selection” of study groups, the “Comparability” of study groups and the ascertainment of the “Outcome” of interest. Specifically, the “Selection” domain evaluates the representativeness of the exposed cohort, the selection of the non-exposed cohort on whether it was drawn from the same community as the exposed cohort, the ascertainment of the exposure, and the demonstration that the outcome of interest was not present at the start of the study. The “Comparability” domain evaluates whether the exposed and non-exposed cohorts are matched in terms of design and whether important confounders have been used for adjustment in the analyses. The “Outcome” domain assesses the methods used for adequate outcome confirmation, the follow-up period on providing enough time for outcome to occur and the adequacy of the follow-up of cohorts. By assessing the aforementioned three domains, we calculated the total quality score of each eligible study. Any disagreements regarding risk of bias assessment were discussed with a third author (IIS) and were resolved through consensus.

### Secondary analysis

We performed the secondary analysis using individual patient-level data from three randomized controlled trials conducted in North America by the Prevention and Early Treatment of Acute Lung Injury (PETAL) Network; namely, VIOLET [18], CLOVERS [19] and ASTER [20]. All three trials [18–20], published between 2019 and 2024, enrolled adult critically ill patients (≥ 18 years old) who had at least one risk factor for development of ARDS. Adjudication of development of ARDS was consistent across trials [18–20]. Specifically, in all three trials [18–20], ARDS was defined as acute hypoxemia [i.e., a partial pressure of arterial oxygen to fraction of inspired oxygen ratio (PaO_2_:FiO_2_) ≤ 300 mmHg], accompanied by bilateral infiltrates on chest radiography, not fully explained by cardiac failure or fluid overload [21]. As previously [22–24], we were granted access to data from each trial [18–20] through the Biologic Specimen and Data Repository Information Coordinating Center of the National Heart, Lung and Blood Institute. Since we would receive data in de-identified form, the Institutional Review Board waived the need of informed consent and approved the study (501/8–11−2024).

### Subject selection, data collection and outcome in the secondary analysis

For inclusion in the secondary analysis, we only considered participants enrolled in the three trials [18–20] with available data on platelet count and no evidence of ARDS at baseline; i.e., at the day of trial enrollment. As baseline platelet count, we considered the most recent platelet count prior to trial enrollment.

From participants who met the abovementioned eligibility criteria (i.e., they had available platelet count data but not ARDS at baseline), we collected the following data: sex, age, body mass index, race, comorbidities, primary risk factor for ARDS, non-pulmonary organ failures, baseline non-coagulation Sequential Organ Failure Assessment (SOFA) score, white blood cell count, hematocrit and plasma intereleukin-6 levels. As previously [25, 26], comorbidities included diabetes mellitus, chronic lung disease, chronic kidney disease, liver disease, hematologic malignancy (i.e., malignant lymphomas and leukemias), solid tumor, prior myocardial infarction and congestive heart failure. Non-pulmonary organ failures (namely, cardiovascular, renal and hepatic failure) were defined as previously [26, 27]. Regarding coagulation failure, data on platelet count were collected. The baseline non-coagulation SOFA score was calculated as the total baseline SOFA score minus the coagulation-specific SOFA score (which is based on platelet count); if data on an organ-specific SOFA score were unavailable, it was considered to be normal.

The outcome of interest in the secondary analysis was development of ARDS. Development of ARDS should have occurred within seven days after trial enrollment, which was the longest follow-up period for ARDS development across the three trials [18–20].

### Statistical analysis

For the meta-analysis, we presented pooled dichotomous effect measures as risk ratios (RR) with 95% confidence intervals (CI), calculated using the Mantel-Haenszel random effects model, as previously [28]. We assessed statistical heterogeneity using the I^2^ statistic and classified it as non-important, moderate, substantial or considerable for values of I^2^ < 40%, < 60%, < 90% and ≥ 90%, respectively. We used Review Manager 5.4 software (Cochrane Collaboration) for data synthesis.

For the secondary analysis, we carried out unadjusted and adjusted binary logistic regression analyses using development of ARDS as the dependent variable and platelet count as an independent variable. Platelet count was treated both as a dichotomous variable (using a cut-off of 100,000 platelets/µL based on previous relevant literature) [29–33] and as a continuous variable (in increments of 10,000/µL). For the adjusted analyses, we selected covariates (namely, sex, age, extrapulmonary sepsis as a risk factor for ARDS, hematologic malignancy, baseline non-coagulation SOFA score and trial) based on previous relevant literature [34, 35] and our clinical judgement. We repeated the abovementioned adjusted analyses for each trial separately [18–20]. We also repeated the abovementioned adjusted analyses by adding either baseline white blood cell count and hematocrit or plasma interleukin-6 levels as covariates in the model.

In order to account for the competing risk of death throughout the 7-day period of follow-up for ARDS development, we conducted competing risks analyses [36]. To this end, we created adjusted Fine-Gray competing risk regression models and generated subdistribution hazard ratios (sHR) [37]. Additionally, we created adjusted cause-specific Cox regression models and generated cause-specific hazard ratios (csHR). In line with our previous analyses, the estimates were adjusted for sex, age, extrapulmonary sepsis as a risk factor for ARDS, hematologic malignancy, baseline non-coagulation SOFA score and trial. As competing event we considered death within seven days from trial enrollment. We repeated this approach separately, treating platelets first as a dichotomous variable (using a cut-off of 100,000/µL) and then as a continuous variable (in increments of 10,000/µL). For the cause-specific Cox regression model, we assessed the linearity of the continuous variables (namely, platelet count, age and baseline non-coagulation SOFA score) using the Martingale residuals and evaluated for potential violations of the assumption of proportional hazards using the Schoenfeld residuals [38]. Finally, we generated adjusted cause-specific cumulative incidence curves to compare the risk of ARDS development between patients with versus without thrombocytopenia (defined as < 100,000 platelets/µL).

In order to visualize the expected potential risk of developing ARDS across various values of platelet count (as a continuous variable), we conducted propensity score weighting using generalized linear models and we generated an exposure-response curve. Initially, we estimated entropy balancing weights that balance the covariates used in the model with respect to the independent variable of interest (namely, platelet count) [39]. After creating weights based on the propensity scores for all the potential covariates (namely, sex, age, extrapulmonary sepsis as a risk factor for ARDS, hematologic malignancy, baseline non-coagulation SOFA score and trial), we assessed the adequacy of balance achieved for each covariate and the effective sample size [40]. We confirmed that all covariates were sufficiently balanced, since the standardized mean difference for the continuous variables and the difference in proportions for the binary variables were < 0.05 [41]. Next, we compared the nested models which included a linear versus spline transformation of the platelet count variable using the likelihood ratio test [42] and the Akaike Information Criterion [43]. We performed comparisons of nested models for both the unweighted and weighted adjusted binary logistic regression models. We also assessed the assumption of linear relationship between the continuous independent variables included in the adjusted model and the logit transformation of the outcome using the Box-Tidwell test [44]. We selected the splines regression model as the optimal model based on the abovementioned tests [42–44]. Subsequently, we fitted a generalized linear model which incorporated a natural cubic splines transformation for the platelet count variable, with four degrees of freedom, and we performed weighted g-computation to estimate the expected potential risk of ARDS development under a representative set of platelet count values. We further assessed the association between platelet count and development of ARDS across the spectrum of platelet count values relative to a reference value. To this end, we performed an adjusted binary logistic regression analysis and we considered as reference value a platelet count of 200,000/µL, which reflected the median of our study population. Adjustments included sex, age, extrapulmonary sepsis as a risk factor for ARDS, hematologic malignancy, baseline non-coagulation SOFA score and trial, as previously. We utilized a natural cubic splines transformation, with four degrees of freedom, for the platelet count variable.

We presented continuous variables as median with interquartile range (IQR) and categorical variables as percentages. All *p* values were two-sided, and we considered statistical significance at an α level of 0.05. We conducted all statistical analyses using SPSS software version 28.0 (SPSS, Inc., Chicago, IL, USA) and R software version 4.4.1 (R Foundation for Statistical Computing).

## Results

### Meta-analysis of observational studies

**Fig. 1:**
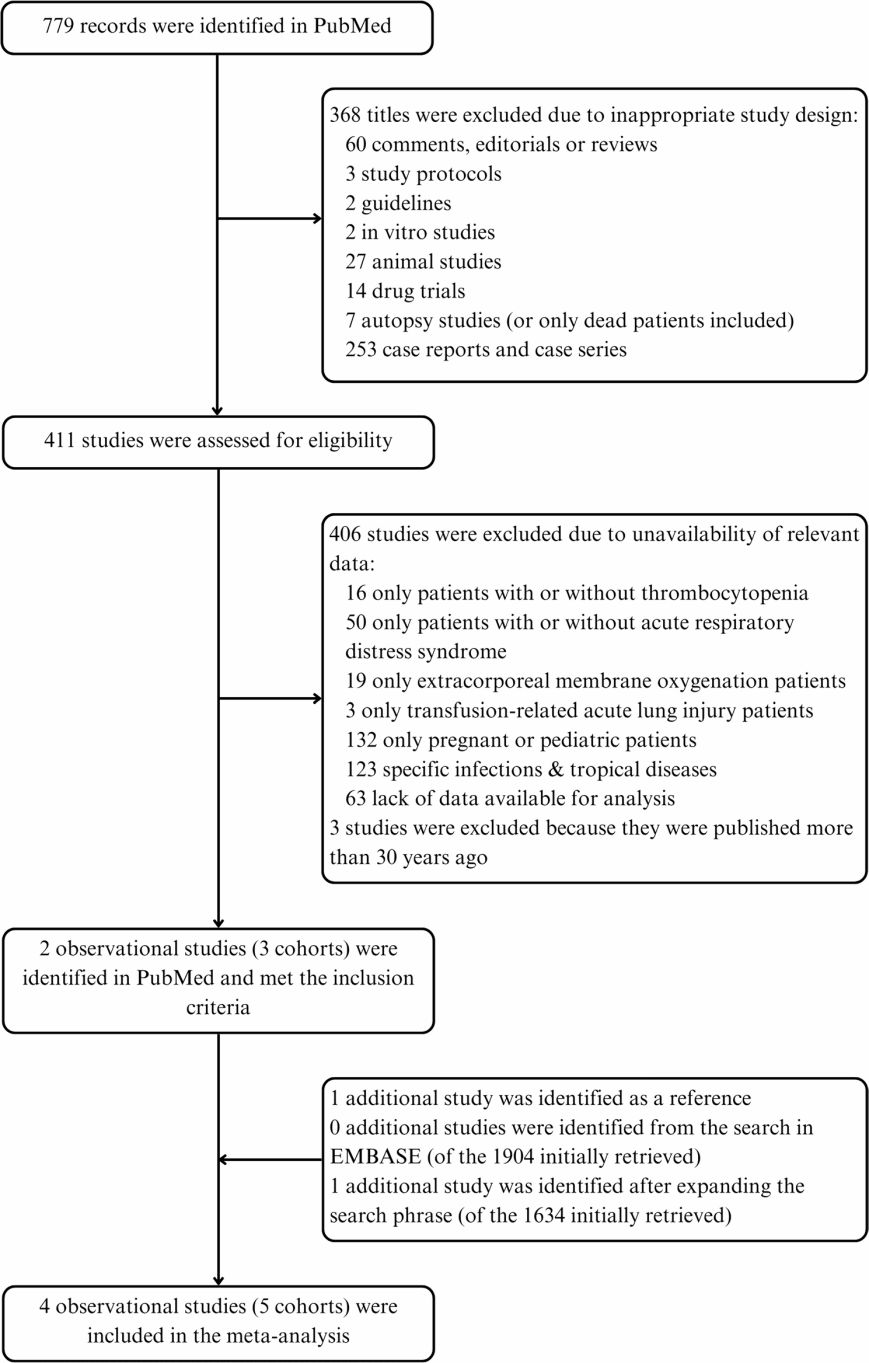
Flow diagram of observational studies included in the systematic review and meta-analysis

Figure 1 depicts the flow diagram for study selection. Of the 779 records initially identified in PubMed, we excluded three relevant studies [29–31] because they were published more than 30 years ago and they did not probably reflect contemporary clinical practice. On the other hand, we incorporated in the meta-analysis the large prospective observational PETAL LOTUS-FRUIT study, for which we got access on data on both the platelet count at baseline and development of ARDS [33]. Therefore, four observational studies (involving five cohorts) were included in the meta-analysis [32–34, 45].

### Characteristics of observational studies included in the meta-analysis

Table 1 shows the characteristics of the four observational studies (five cohorts) [32–34, 45]. To define thrombocytopenia, two studies [32, 33] used a cut-off of 100,000 platelets/µL, one study [34] used a cut-off of 80,000 platelets/µL and one study [45] used a cut-off 125,000 platelets/µL. Three studies used the Berlin definition of ARDS [32, 33, 45]. The most common risk factor for ARDS was sepsis, either pulmonary (pneumonia) or extrapulmonary. Whether the association between thrombocytopenia and development of ARDS persisted after adjustment for covariates was estimated in two studies [34, 45]. Of those, one study excluded patients with hematologic malignancy [34] and the other study excluded patients with malignancy [45].

**Table 1:** Characteristics of the four observational studies (five cohorts) included in the meta-analysis

First Author, country, ref	Year	Study design	Number of included patients, *N*	Definition of thrombocytopenia (platelet count <)	Definition of ARDS	Risk factors for ARDS	Was thrombocytopenia associated with development of ARDS after adjustment for confounders?
Gao, China, 32	2013	Retrospective Multicenter	111	100,000/µL	Berlin definition	Influenza A (H7N9) virus infection	NR
Wang (Beijing cohort), China, 34	2014	Prospective Double-center	178	80,000/µL	American-European Consensus Committee definition	Sepsis, pneumonia, trauma, aspiration, multiple transfusions, pancreatitis	Yes*
Wang (Boston cohort), USA, 34	2014	Prospective Double-center	1983	80,000/µL	American-European Consensus Committee definition	Sepsis, pneumonia, trauma, aspiration, multiple transfusions	Yes*
LOTUS FRUIT, USA, 33	2019	Prospective Multicenter	1167^#^	100,000/µL	Berlin definition	Sepsis, pneumonia, aspiration, shock, trauma, other	NR
Lin, China, 45	2022	Retrospective Multicentre	234	125,000/µL	Berlin definition	Severe acute pancreatitis	Yes**

*Abbreviations: ref* reference, *ARDS* acute respiratory distress syndrome, *NR* not reported, *PaO*_*2*_ partial pressure of arterial oxygen, *FiO*_*2*_ fraction of inspired oxygen

*Wang et al reported adjustment for potential confounders, such as extrapulmonary sepsis. That study excluded patients with hematologic malignancy

**Lin et al reported adjustment for potential confounders, such as partial pressure of arterial oxygen to fraction of inspired oxygen ratio. That study excluded patients with malignancy

^#^Refers to patients without ARDS at baseline

Supplemental Table 1 depicts the assessment for risk of bias among included studies. Bias regarding the “comparability” domain was common, as most studies did not adjust for important confounders.

### Outcome in the meta-analysis of observational studies

**Fig. 2:**
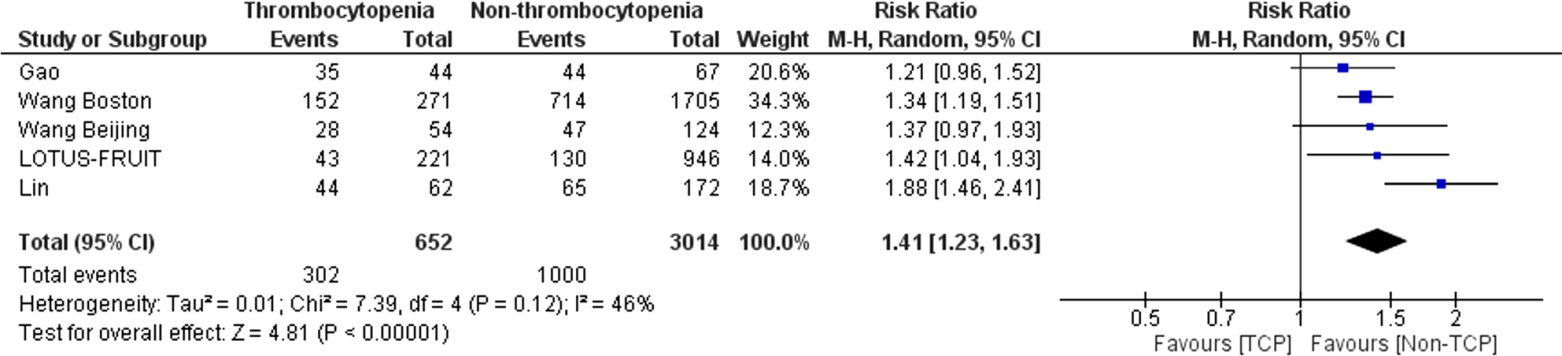
Forest plot of the development of acute respiratory distress syndrome in patients with versus without thrombocytopenia. Pooled risk ratio (RR) and 95% confidence intervals (CI) were calculated using the Mantel-Haenszel random effects model. *Abbreviation*: TCP, thrombocytopenia

Four observational studies, involving five cohorts and a total of 3666 critically ill patients at risk for ARDS, provided data on development of ARDS [32–34, 45]. Figure 2 shows that patients with thrombocytopenia were more likely to develop ARDS than those without thrombocytopenia (46.3% versus 33.2%; RR 1.41, 95% CI 1.23–1.63; *p* < 0.001). Statistical heterogeneity (I^2^ = 46%) was moderate.

### Secondary analysis of three randomized controlled trials

#### Characteristics of participants included in the secondary analysis

Out of the 3370 participants in the three randomized controlled trials [18–20], 2927 subjects had available platelet count data but not ARDS at baseline and, therefore, were included in our secondary analysis. Table 2 and Supplemental Table 2 show the characteristics of the 2927 included participants. Their median age was 60.0 (IQR, 49.0–70.0) years old, 1378 (47.1%) were female, 189 (6.5%) had hematologic malignancy, and 1694 (57.9%) had extrapulmonary sepsis as primary risk factor for ARDS. Median platelet count was 203,000 (137,000–284,000)/µL at baseline. Supplemental Fig. 1 provides histograms with rug strikes depicting distribution of platelet count values among participants in the three randomized controlled trials [18–20]. One hundred and four patients (3.6%) eventually developed ARDS within seven days after trial enrollment. Supplemental Table 3 shows missing data on characteristics of patients included in each of the three randomized controlled trials [18–20]. Complete case analyses were performed.

**Table 2:** Characteristics of patients included in the secondary analysis of three randomized controlled trials

Female sex	1378 (47.1)
Age	60.0 (49.0–70.0)
Body mass index	26.7 (22.5–32.6)
Race	
White	1794 (61.3)
Black or African American	515 (17.6)
Hispanic or Latino	395 (13.5)
Other	102 (3.5)
Comorbidities	
Diabetes mellitus	921 (31.5)
Chronic lung disease	494 (16.9)
Chronic kidney disease	355 (12.1)
Liver disease	304 (10.4)
Hematologic malignancy	189 (6.5)
Solid tumor	458 (15.6)
Prior myocardial infarction	217 (7.4)
Congestive heart failure	387 (13.2)
Primary risk factor for ARDS	
Pneumonia	918 (31.4)
Extrapulmonary sepsis	1694 (57.9)
Organ failure at baseline	
Cardiovascular	1413 (48.3)
Renal	832 (28.4)
Hepatic	344 (11.8)
Non-coagulation SOFA score	3.0 (1.0–5.0)
White blood cell count (/µL)	11,755 (6,952–17,620)
Hematocrit (%)	34.6 (29.2–39.2)
Interleukin-6 (pg/mL)	48.8 (17.1–201.5)
Baseline platelets (/µL)	203,000 (137,000–284,000)
Development of ARDS	104 (3.6)

Data are presented as median (interquartile range) or numbers (percentages)

Data from 2927 subjects at risk for ARDS were considered for the secondary analysis

Data on white blood cell count were available only for VIOLET [18] and CLOVERS [19] trials

Data on hematocrit were available only for CLOVERS [19] trial

Data on plasma interleukin-6 levels were available only for VIOLET [18] and ASTER [20] trials

*Abbreviations:* *ARDS* acute respiratory distress syndrome, *SOFA* Sequential Organ Failure Assessment

### Outcome in the secondary analysis

When we treated platelet count as a dichotomous variable with a cut-off of 100,000/µL to define thrombocytopenia, we found that thrombocytopenia was associated with development of ARDS [2927 patients, 104 events; odds ratio (OR) 1.70, 95% CI 1.05–2.73; *p* = 0.029] in unadjusted analyses. However, after adjustment for sex, age, extrapulmonary sepsis as a primary risk factor for ARDS, hematologic malignancy, baseline non-coagulation SOFA score and trial, thrombocytopenia was not independently associated with development of ARDS (2883 patients, 102 events; OR 1.57, 95% CI 0.95–2.60; *p* = 0.080).

When we treated platelet count as a continuous variable, we found that platelet count was not associated with development of ARDS (2927 patients, 104 events; OR 0.99, 95% CI 0.98–1.01; *p* = 0.450) in unadjusted analyses. Consistently, even after adjustment for sex, age, extrapulmonary sepsis as a primary risk factor for ARDS, hematologic malignancy, baseline non-coagulation SOFA score and trial, platelet count (as a continuous variable) was not independently associated with development of ARDS (2883 patients, 102 events; OR 1.00, 95% CI 0.98–1.02; *p* = 0.981). Supplemental Table 4 shows the results of the corresponding unadjusted and adjusted analyses separately for each randomized controlled trial [18–20]. As shown in Supplemental Table 5, the finding of the main analysis persisted (i.e., platelet count was not independently associated with development of ARDS) when we repeated the analyses by adding either baseline white blood cell count and hematocrit or plasma interleukin-6 levels as covariates in the model.

The finding of the main analysis persisted even after accounting for the competing risk of death throughout the 7-day period of follow-up for ARDS development. Specifically, in the adjusted Fine-Gray competing risk regression models, we found that platelet count [either analyzed as a dichotomous variable (sHR 1.52, 95% CI 0.94–2.44; *p* = 0.09) (Supplemental Table 6) or as s continuous variable (sHR 1.00, 95% CI 0.98–1.02; *p* = 0.99) (Supplemental Table 7)] was not independently associated with ARDS development. Consistently, in the adjusted cause-specific Cox regression models, we found that platelet count [either analyzed as a dichotomous variable (csHR 1.57, 95% CI 0.97–2.54; *p* = 0.07) (Supplemental Table 8) or as s continuous variable (csHR 1.00, 95% CI 0.98–1.02; *p* = 0.95) (Supplemental Table 9)] was not independently associated with ARDS development. For both the abovementioned adjusted cause-specific Cox regression models, plots of Martingale residuals and Schoenfeld residuals are shown in Supplemental Figs. 2–5. Finally, Supplemental Fig. 6 shows adjusted cause-specific cumulative incidence curves comparing the risk of ARDS development between patients with versus without thrombocytopenia (defined as < 100,000 platelets/µL).

**Fig. 3:**
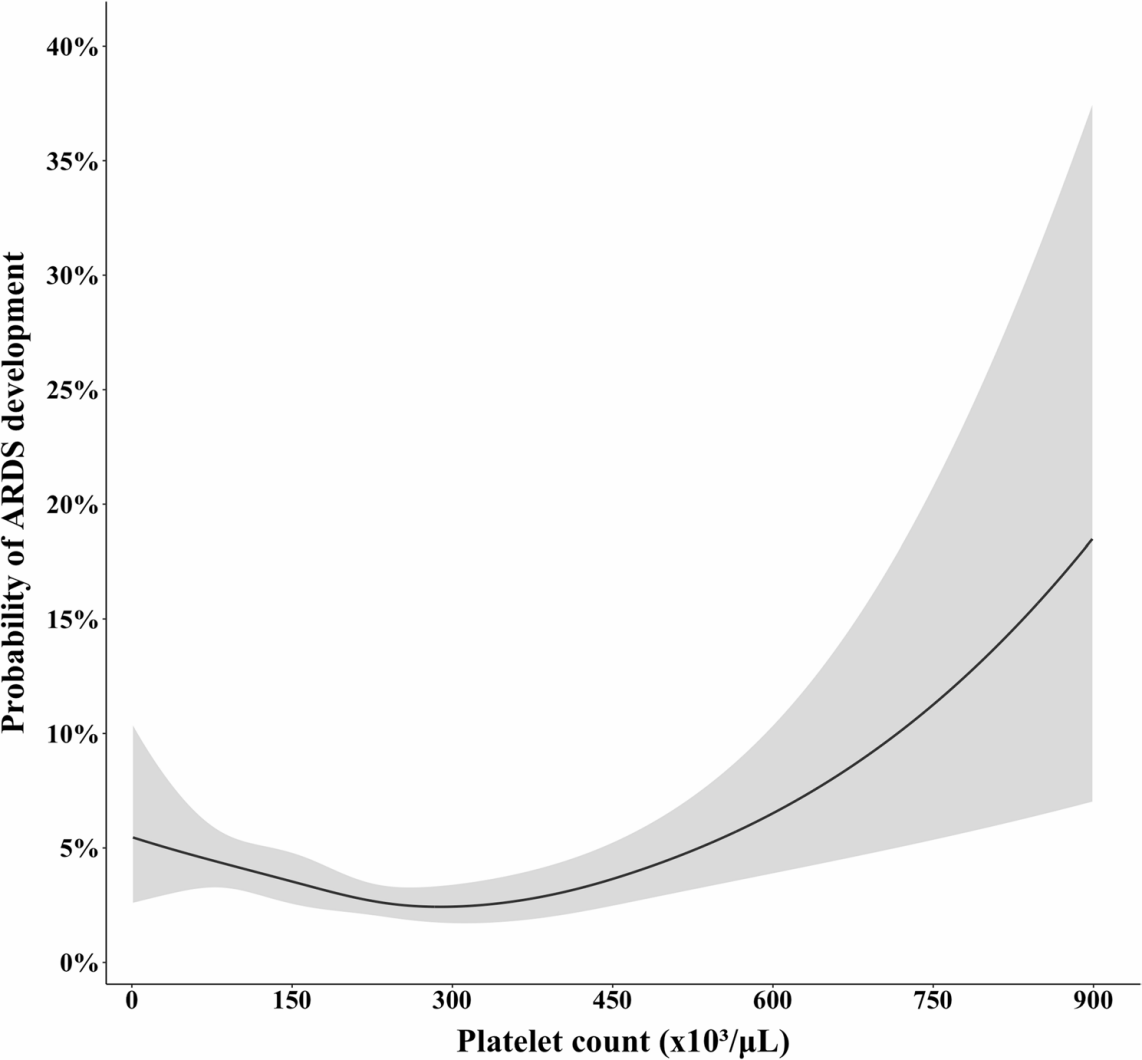
Association between platelet count as a continuous variable and development of acute respiratory distress syndrome in the secondary analysis of three randomized controlled trials. ARDS, acute respiratory distress syndrome

In order to visualize the association between platelet count (as a continuous variable) and development of ARDS, we initially compared models using the likelihood ratio test and we found that the model including the spline transformation of platelet count provided a significantly better fit than the linear model (*p* = 0.003 for the comparison of unweighted models, *p* = 0.004 for the comparison of weighted models). Consistently, the Akaike Information Criterion further supported improved model fit after incorporating splines, for both the unweighted and weighted models. The Box-Tidwell test also indicated that the linearity in the logit was violated for the platelet count variable (*p* < 0.001). Taken together, the abovementioned tests (namely, likelihood ratio test, Akaike Information Criterion and Box-Tidwell test) indicated that the association between platelet count and ARDS development was non-linear. Figure 3 shows that the association between platelet count (as a continuous variable) and development of ARDS was non-linear and appeared U-shaped. Consistently, a similar U-shape was noted after visualizing the adjusted odds ratios for ARDS development across different platelet count values, using 200,000/µL as a reference value (Supplemental Fig. 7). The corresponding ORs and 95% CI are shown in Supplemental Table 10.

## Discussion

By accumulating evidence from four observational studies (five cohorts) involving 3666 critically ill patients at risk for ARDS, we found that thrombocytopenia was associated with development of ARDS. However, by taking advantage of individual patient-level data from 2927 critically ill patients enrolled in three recent, large and high-quality randomized controlled trials, we found that thrombocytopenia was not independently associated with development of ARDS after adjustment for important confounders, such as non-coagulation SOFA score. The association between platelet count (treated as a continuous variable) and development of ARDS was non-linear and appeared U-shaped. These findings suggest that thrombocytopenia may serve as a marker of severe systemic illness and its association with ARDS may not represent a mechanistic relationship.

Our systematic review and meta-analysis of observational studies may constitute the most comprehensive effort to synthesize clinical evidence pertaining to the association between thrombocytopenia and development of ARDS. We found that patients with thrombocytopenia were more likely to develop ARDS than those without thrombocytopenia. Yet, the most important finding of the systematic review may be the exposure of the limitations of existing evidence in the field. Indeed, we excluded three small studies which were published more than 30 years ago [29–31]. Those studies were probably outdated because supportive care of critically ill patients substantially improved over the last years resulting in fewer cases of ARDS [46]. With regard to the studies eventually included in the systematic review [32–34, 45], they had heterogenous thrombocytopenia thresholds (ranging from 80,000 to 125,000 platelets/µL). Also, most of them did not perform adjustments for important confounders, such as extrapulmonary sepsis. Even the one study, which adjusted for extrapulmonary sepsis, excluded patients with hematologic malignancy [34]. Taken together, our systematic review enabled us to identify limitations in the existing evidence which we endeavoured to address by subsequently performing a secondary analysis.

Our subsequent secondary analysis of randomized controlled trials indeed addressed many of the abovementioned limitations of existing evidence. All randomized controlled trials were large (enrolling a total of almost 3,000 critically ill patients) and were published during the last five years (thus, reflecting contemporary supportive care of critically ill patients at risk for ARDS) [18–20]. Also, given that we had access to high-quality, granular, individual patient-level data from these three randomized controlled trials [18–20], we were able to explore the role of potential confounders on the association between thrombocytopenia and development of ARDS. We found that, after adjustment for confounders, such as sex, age, extrapulmonary sepsis as a primary risk factor for ARDS, hematologic malignancy, baseline non-coagulation SOFA score and trial, thrombocytopenia was not independently associated with development of ARDS.

For our abovementioned analyses, we used a widespread definition of thrombocytopenia (namely, < 100,000 platelets/µL) and therefore we treated platelet count as a dichotomous variable. Treating platelet count as a dichotomous variable, albeit common in the literature [47, 48], may not be optimal from a statistical point of view. To address this concern, we also treated platelet count as a continuous variable. The latter analyses were indeed informative; they suggested that the association between platelet count (as a continuous variable) and development of ARDS may be non-linear (as rigorously quantified by using likelihood ratio test, Akaike Information Criterion and Box-Tidwell test) and appeared U-shaped. A U-shaped association between platelet count and mortality has been previously shown for the general population [49]. The present study may be novel in suggesting that a U-shape might also describe the association between platelet count and development of ARDS among critically ill patients.

A notable strength of our study may be its dual design which combined a PRISMA-registered, study-level meta-analysis with an individual patient-level secondary analysis of three contemporary PETAL Network randomized controlled trials. That being said, our study has limitations. First, a limitation of the secondary analysis is that the numbers of patients with higher platelet count were limited and varied across the three randomized controlled trials [18–20]. This fact (reflected in the relevant wide confidence intervals of Fig. 3) did not allow us for inferring as to whether higher platelet count may be associated with development of ARDS. However, exploring such an association was beyond the scope of this study which focused on thrombocytopenia. Thrombocytopenia was not uncommon among participants in all three randomized controlled trials [18–20] and this adequacy of granular relevant data may enhance the robustness of our main finding (namely, thrombocytopenia is not independently associated with development of ARDS). Second, although we focused on thrombocytopenia at baseline, we acknowledge that thrombocytopenia is often a dynamic rather than fixed measurement. Third, due to unavailability of relevant data, we could not adjust for markers of coagulation, such as prothrombin time or international normalized ratio, to identify those patients who have thrombocytopenia due to disseminated intravascular coagulation and to better understand whether it is thrombocytopenia per se that is associated with the development of ARDS, or just coagulation dysfunction globally.

## Conclusions

In conclusion, our comprehensive meta-analysis of four observational studies (five cohorts) involving over 3500 critically ill patients suggests that thrombocytopenia was associated with development of ARDS. However, our subsequent secondary analysis of individual patient-level data from almost 3000 critically ill patients enrolled in three randomized controlled trial suggests that thrombocytopenia was not independently associated with development of ARDS after adjustment for important confounders, including severity of illness. The secondary analysis also suggests that the association between platelet count (treated as a continuous variable) and development of ARDS might be U-shaped. Based on these findings, clinicians may view thrombocytopenia as a marker of severe systemic illness, which may be indirectly (i.e., not mechanistically) associated with ARDS.

## Supplementary Information

Supplementary Material 1.

## Data Availability

Data which this secondary analysis was based on are available through the Biologic Specimen and Data Repository Information Coordinating Center of the National Heart, Lung, and Blood Institute (https://biolincc.nhlbi.nih.gov/home/).
