# Supplementary material for: Association between thrombocytopenia and development of acute respiratory distress syndrome
Source: Respir Res. 2026 Jan 13;27:46. doi: 10.1186/s12931-025-03444-x (PMC12888621; doi:10.1186/s12931-025-03444-x)

**Online Data Supplement**

**Association between thrombocytopenia and development of acute respiratory distress syndrome**

Elpida Charalampaki,^*^ Konstantinos Gkirgkiris,^*^ David R. Price, Eleni Papoutsi, Georgia M. Minatsi, Georgia Dimopoulou, Stylianos E. Orfanos, Ioanna Dimopoulou, Anastasia Kotanidou, Ilias I. Siempos

*These two authors contributed equally.

| Supplemental Table 1. Risk of bias assessment of the four observational studies (five cohorts) included in the meta-analysis. | | | | | | | | | | |
| --- | --- | --- | --- | --- | --- | --- | --- | --- | --- | --- |
| Study | Selection | | | | Comparability | Outcome | | |  |  |
| Author | Representativeness of the exposed cohort (Max:**🟑**) | Selection of the non-exposed cohort (Max:**🟑**) | Ascertainment of exposure (Max:**🟑**) | Demonstration that outcome of interest was not present at start of study (Max:**🟑**) | Comparability of cohorts on the basis of the design or analysis (Max:**🟑🟑**) | Assessment of outcome  (Max:**🟑**) | Was follow-up long enough for outcomes to occur (Max:**🟑**) | Adequacy of follow up of cohorts (Max:**🟑**) | Total quality score  (out of 9) |  |
| Gao | **🟑** | **🟑** | **🟑** | - | - | **🟑** | **🟑** | - | **🟑** **🟑** **🟑** **🟑** **🟑** (5) |  |
| Wang (Beijing cohort) | **🟑** | **🟑** | **🟑** | - | - | **🟑** | **🟑** | **🟑** | **🟑** **🟑** **🟑** **🟑** **🟑** **🟑** (6) |  |
| Wang (Boston cohort) | **🟑** | **🟑** | **🟑** | - | - | **🟑** | **🟑** | **🟑** | **🟑** **🟑** **🟑** **🟑** **🟑** **🟑** (6) |  |
| LOTUS FRUIT | **🟑** | **🟑** | **🟑** | **🟑** | - | **🟑** | **🟑** | **🟑** | **🟑** **🟑** **🟑** **🟑** **🟑** **🟑** **🟑** (7) |  |
| Lin | **🟑** | **🟑** | **🟑** | **🟑** | - | **🟑** | **🟑** | **🟑** | **🟑** **🟑** **🟑** **🟑** **🟑** **🟑** **🟑** (7) |  |

| **Supplemental Table 2. Characteristics of patients included in each of the three randomized controlled trials of the secondary analysis.** | | | |
| --- | --- | --- | --- |
| **Variable** | **VIOLET  (n=1036)** | **CLOVERS**  **(n=1490)** | **ASTER  (n=401)** |
| Female sex | 469 (45.3) | 708 (47.5) | 201 (50.1) |
| Age | 58.0 (44.0 – 67.0) | 61.0 (49.0 – 70.0) | 66.0 (56.0 – 76.0) |
| Body mass index | 28.0 (23.3 – 34.6) | 25.8 (22.2 – 30.7) | 27.7 (22.2 – 33.7) |
| Race |  |  |  |
| White | 610 (58.9) | 942 (63.2) | 242 (60.3) |
| Black or African American | 218 (21.0) | 228 (15.3) | 69 (17.2) |
| Hispanic or Latino | 120 (11.6) | 219 (14.7) | 56 (14.0) |
| Other | 28 (2.7) | 49 (3.3) | 25 (6.2) |
| Comorbidities |  |  |  |
| Diabetes mellitus | 354 (34.2) | 420 (28.2) | 147 (36.7) |
| Chronic lung disease | 205 (19.8) | 230 (15.4) | 59 (14.7) |
| Chronic kidney disease | 164 (15.8) | 151 (10.1) | 40 (10.0) |
| Liver disease | 110 (10.6) | 178 (11.9) | 16 (4.0) |
| Hematologic malignancy | 46 (4.4) | 112 (7.5) | 31 (7.7) |
| Solid tumor | 113 (10.9) | 288 (19.3) | 57 (14.2) |
| Prior myocardial infarction | 92 (8.9) | 97 (6.5) | 28 (7.0) |
| Congestive heart failure | 160 (15.4) | 164 (11.0) | 63 (15.7) |
| Primary risk factor for ARDS |  |  |  |
| Pneumonia | 353 (34.1) | 402 (27.0) | 163 (40.6) |
| Extrapulmonary sepsis | 368 (35.5) | 1088 (73.0) | 238 (59.4) |
| Organ failure at baseline |  |  |  |
| Cardiovascular | 384 (37.1) | 715 (48.0) | 314 (78.3) |
| Renal | 306 (29.5) | 419 (28.1) | 107 (26.7) |
| Hepatic | 155 (15.0) | 164 (11.0) | 25 (6.2) |
| Non-coagulation SOFA score | 4.0 (2.0 – 6.0) | 2.0 (1.0 – 4.0) | 5.0 (4.0 – 6.0) |
| White blood cell count (/μL) | 11,500  (7,000 – 17,850) | 12,000  (6,900 – 17,500) | NA |
| Hematocrit (%) | NA | 34.6 (29.2 – 39.2) | NA |
| Interleukin-6 (pg/mL) | 88.8 (24.5 – 356.7) | NA | 34.9 (14.6 – 127.4) |
| Baseline platelets (/μL) | 190,000  (128,000 – 264,000) | 215,000  (142,000 – 303,000) | 203,000  (131,500 – 278,500) |
| Development of ARDS | 44 (4.2) | 38 (2.6) | 22 (5.5) |

*Abbreviations:* ARDS, acute respiratory distress syndrome; SOFA, Sequential Organ Failure Assessment; NA, not available

Data are presented as median (interquartile range) or numbers (percentages).

Data from 2927 subjects at risk for ARDS were considered for the secondary analysis.

Data on white blood cell count were only available for VIOLET and CLOVERS trials.

Data on hematocrit were only available for CLOVERS trial.

Data on plasma interleukin-6 levels were only available for VIOLET and ASTER trials.

| **Supplemental Table 3. Missing data on characteristics of patients included in each of the three randomized controlled trials of the secondary analysis.** | | | | |
| --- | --- | --- | --- | --- |
| **Variable** | **Total**  **(n=2927)** | **VIOLET**  **(n=1036)** | **CLOVERS**  **(n=1490)** | **ASTER**  **(n=401)** |
| Female sex | 0 (0.0) | 0 (0.0) | 0 (0.0) | 0 (0.0) |
| Age | 1 (0.0) | 0 (0.0) | 0 (0.0) | 1 (0.2) |
| Body mass index | 73 (2.5) | 25 (2.4) | 47 (3.2) | 1 (0.2) |
| Race | 121 (4.1) | 60 (5.8) | 52 (3.5) | 9 (2.2) |
| Comorbidities |  |  |  |  |
| Diabetes mellitus | 42 (1.4) | 29 (2.8) | 13 (0.9) | 0 (0.0) |
| Chronic lung  disease | 42 (1.4) | 29 (2.8) | 13 (0.9) | 0 (0.0) |
| Chronic kidney  disease | 42 (1.4) | 29 (2.8) | 13 (0.9) | 0 (0.0) |
| Liver disease | 42 (1.4) | 29 (2.8) | 13 (0.9) | 0 (0.0) |
| Hematologic  malignancy | 42 (1.4) | 29 (2.8) | 13 (0.9) | 0 (0.0) |
| Solid tumor | 42 (1.4) | 29 (2.8) | 13 (0.9) | 0 (0.0) |
| Prior myocardial  infarction | 42 (1.4) | 29 (2.8) | 13 (0.9) | 0 (0.0) |
| Congestive heart  failure | 42 (1.4) | 29 (2.8) | 13 (0.9) | 0 (0.0) |
| Primary risk factor  for ARDS |  |  |  |  |
| Pneumonia | 0 (0.0) | 0 (0.0) | 0 (0.0) | 0 (0.0) |
| Extrapulmonary  sepsis | 0 (0.0) | 0 (0.0) | 0 (0.0) | 0 (0.0) |
| Organ failure at  baseline |  |  |  |  |
| Cardiovascular | 0 (0.0) | 0 (0.0) | 0 (0.0) | 0 (0.0) |
| Renal | 0 (0.0) | 0 (0.0) | 0 (0.0) | 0 (0.0) |
| Hepatic | 0 (0.0) | 0 (0.0) | 0 (0.0) | 0 (0.0) |
| Non-coagulation SOFA score | 1 (0.0) | 0 (0.0) | 0 (0.0) | 1 (0.2) |
| White blood cells | 425 (14.5) | 17 (1.6) | 7 (0.5) | 401 (100.0) |
| Hematocrit | 1444 (49.3) | 1036 (100.0) | 7 (0.5) | 401 (100.0) |
| Interleukin-6 | 2300 (78.6) | 800 (77.2) | 1490 (100.0) | 10 (2.5) |

*Abbreviations:* ARDS, acute respiratory distress syndrome; SOFA, Sequential Organ Failure Assessment

Data are presented as number of patients (%).

| **Supplemental Table 4. Association between platelet count (treated as a dichotomous or as a continuous variable) and development of ARDS in unadjusted and adjusted analyses in each of the three randomized controlled trials (VIOLET, CLOVERS and ASTER) of the secondary analysis.** | | | | | |
| --- | --- | --- | --- | --- | --- |
| **Trial** | **Number of cases** | **Number of events** | **Odds ratio** | **95% Confidence Intervals** | **p value** |
| **VIOLET** |  |  |  |  |  |
| Platelet count as dichotomous variable |  |  |  |  |  |
| Unadjusted | 1036 | 44 | 2.373 | 1.213 – 4.640 | 0.012 |
| Adjusted | 1007 | 42 | 2.106 | 1.021 – 4.344 | 0.044 |
| Platelet count as continuous variable |  |  |  |  |  |
| Unadjusted | 1036 | 44 | 0.952 | 0.922 – 0.984 | 0.003 |
| Adjusted | 1007 | 42 | 0.958 | 0.926 – 0.991 | 0.014 |
| **CLOVERS** |  |  |  |  |  |
| Platelet count as dichotomous variable |  |  |  |  |  |
| Unadjusted | 1490 | 38 | 1.709 | 0.772 – 3.781 | 0.186 |
| Adjusted | 1477 | 38 | 1.736 | 0.756 – 3.985 | 0.193 |
| Platelet count as continuous variable |  |  |  |  |  |
| Unadjusted | 1490 | 38 | 1.012 | 0.991 – 1.034 | 0.271 |
| Adjusted | 1477 | 38 | 1.018 | 0.995 – 1.041 | 0.131 |
| **ASTER** |  |  |  |  |  |
| Platelet count as dichotomous variable |  |  |  |  |  |
| Unadjusted | 401 | 22 | 0.532 | 0.121 – 2.334 | 0.403 |
| Adjusted | 399 | 22 | 0.534 | 0.116 – 2.456 | 0.421 |
| Platelet count as continuous variable |  |  |  |  |  |
| Unadjusted | 401 | 22 | 1.020 | 0.989 – 1.052 | 0.214 |
| Adjusted | 399 | 22 | 1.021 | 0.987 – 1.055 | 0.227 |

*Abbreviations:* ARDS, acute respiratory distress syndrome

In adjusted analyses, adjustments were made for sex, age, extrapulmonary sepsis as a primary risk factor for ARDS, hematologic malignancy, baseline non-coagulation Sequential Organ Failure Assessment (SOFA) score and trial.

For the analyses treating platelet count as a continuous variable, increments of 10,000 platelets/μL were considered.

| **Supplemental Table 5. Association between platelet count (treated as a dichotomous or as a continuous variable) and development of ARDS in adjusted analyses that incorporate either baseline white blood cell count and hematocrit or plasma interleukin-6 levels as covariates in the model*.** | | | | | |
| --- | --- | --- | --- | --- | --- |
|  | **Number of cases** | **Number of events** | **Odds ratio** | **95% Confidence Intervals** | **p value** |
| **White blood cell count and hematocrit**** | | | | | |
| Platelet count as dichotomous variable | 1470 | 38 | 2.075 | 0.875 – 4.918 | 0.098 |
| Platelet count as continuous variable | 1470 | 38 | 1.014 | 0.990 – 1.038 | 0.253 |
| **Interleukin-6***** | | | | | |
| Platelet count as dichotomous variable | 620 | 33 | 1.021 | 0.367 – 2.844 | 0.968 |
| Platelet count as continuous variable | 620 | 33 | 1.000 | 0.970 – 1.031 | 0.985 |

*Abbreviations:* ARDS, acute respiratory distress syndrome

For the analyses treating platelet count as a continuous variable, increments of 10,000 /μL were utilized.

*The model included the following covariates: sex, age, extrapulmonary sepsis as a primary risk factor for ARDS, hematologic malignancy, baseline non-coagulation Sequential Organ Failure Assessment (SOFA) score and trial. In that model, we incorporated either white blood cell count and hematocrit or plasma interleukin-6 levels as covariates.

**Data on both white blood cell count and hematocrit were only available for CLOVERS trial.

***Data on plasma interleukin-6 levels were only available for VIOLET and ASTER trials.

| **Supplemental Table 6. Adjusted Fine-Gray subdistribution hazard model estimates to isolate the contribution of platelet count (treated as dichotomous variable) to ARDS development and death within 7 days from trial enrollment (competing event) after adjusting for** **sex, age, extrapulmonary sepsis as a primary risk factor for ARDS, hematologic malignancy, baseline non-coagulation SOFA score and trial.** | | | | | | |
| --- | --- | --- | --- | --- | --- | --- |
|  | **ARDS development** | | | **Death** | | |
| **Variable** | **sHR** | **95% Confidence**  **Intervals** | **p value** | **sHR** | **95% Confidence**  **Intervals** | **p value** |
| Platelet count | 1.52 | 0.94 – 2.44 | 0.09 | 1.83 | 1.28 – 2.61 | <0.001 |
| Sex | 1.06 | 0.72 – 1.58 | 0.75 | 1.04 | 0.76 – 1.42 | 0.81 |
| Age | 1.00 | 0.98 – 1.01 | 0.33 | 1.03 | 1.02 – 1.05 | <0.001 |
| Extrapulmonary sepsis* | 0.36 | 0.23 – 0.55 | <0.001 | 1.01 | 0.73 – 1.40 | 0.95 |
| Hematologic malignancy | 0.69 | 0.27 – 1.77 | 0.44 | 1.44 | 0.85 – 2.42 | 0.17 |
| Baseline non-  coagulation SOFA score | 1.18 | 1.10 – 1.25 | <0.001 | 1.33 | 1.26 – 1.40 | <0.001 |
| VIOLET | Ref | Ref | Ref | Ref | Ref | Ref |
| CLOVERS | 1.16 | 0.70 – 1.91 | 0.56 | 1.99 | 1.32 – 3.01 | 0.001 |
| ASTER | 1.64 | 0.95 – 2.84 | 0.08 | 1.32 | 0.82 – 2.12 | 0.25 |

*Abbreviations:* ARDS, acute respiratory distress syndrome; SOFA, Sequential Organ Failure Assessment; sHR, subdistribution hazard ratio; Ref, reference

Platelet count was treated as dichotomous variable using a cut-off of 100,000/μL.

*Versus pulmonary sepsis or no sepsis.

| **Supplemental Table 7. Adjusted Fine-Gray subdistribution hazard model estimates to isolate the contribution of platelet count (treated as continuous variable) to ARDS development and death within 7 days from trial enrollment (competing event) after adjusting for** **sex, age, extrapulmonary sepsis as a primary risk factor for ARDS, hematologic malignancy, baseline non-coagulation SOFA score and trial.** | | | | | | |
| --- | --- | --- | --- | --- | --- | --- |
|  | **ARDS development** | | | **Death** | | |
| **Variable** | **sHR** | **95% Confidence**  **Intervals** | **p value** | **sHR** | **95% Confidence**  **Intervals** | **p value** |
| Platelet count | 1.00 | 0.98 – 1.02 | 0.99 | 1.00 | 0.98 – 1.01 | 0.46 |
| Sex | 1.06 | 0.72 – 1.57 | 0.77 | 1.04 | 0.76 – 1.43 | 0.79 |
| Age | 1.00 | 0.98 – 1.01 | 0.34 | 1.03 | 1.02 – 1.05 | <0.001 |
| Extrapulmonary sepsis* | 0.36 | 0.23 – 0.56 | <0.001 | 1.02 | 0.73 – 1.41 | 0.92 |
| Hematologic malignancy | 0.80 | 0.32 – 2.00 | 0.63 | 1.70 | 1.01 – 2.86 | 0.05 |
| Baseline non-  coagulation SOFA score | 1.18 | 1.11 – 1.26 | <0.001 | 1.33 | 1.27 – 1.41 | <0.001 |
| VIOLET | Ref | Ref | Ref | Ref | Ref | Ref |
| CLOVERS | 1.16 | 0.70 – 1.89 | 0.57 | 2.01 | 1.32 – 3.04 | 0.001 |
| ASTER | 1.64 | 0.96 – 2.82 | 0.07 | 1.36 | 0.85 – 2.18 | 0.20 |

*Abbreviations:* ARDS, acute respiratory distress syndrome; SOFA, Sequential Organ Failure Assessment; sHR, subdistribution hazard ratio; Ref, reference

Platelet count was treated as continuous variable using increments of 10,000/μL.

*Versus pulmonary sepsis or no sepsis.

| **Supplemental Table 8. Adjusted cause-specific Cox regression** **model estimates to isolate the contribution of platelet count (treated as dichotomous variable) to ARDS development and death within 7 days from trial enrollment (competing event) after adjusting for** **sex, age, extrapulmonary sepsis as a primary risk factor for ARDS, hematologic malignancy, baseline non-coagulation SOFA score and trial.** | | | | | | |
| --- | --- | --- | --- | --- | --- | --- |
|  | **ARDS development** | | | **Death** | | |
| **Variable** | **csHR** | **95% Confidence**  **Intervals** | **p value** | **csHR** | **95% Confidence**  **Intervals** | **p value** |
| Platelet count | 1.57 | 0.97 – 2.54 | 0.07 | 1.88 | 1.32 – 2.69 | <0.001 |
| Sex | 1.07 | 0.73 – 1.59 | 0.72 | 1.06 | 0.78 – 1.44 | 0.72 |
| Age | 1.00 | 0.98 – 1.01 | 0.48 | 1.03 | 1.02 – 1.05 | <0.001 |
| Extrapulmonary sepsis* | 0.35 | 0.23 – 0.54 | <0.001 | 0.97 | 0.71 – 1.34 | 0.87 |
| Hematologic malignancy | 0.68 | 0.27 – 1.70 | 0.41 | 1.42 | 0.83 – 2.43 | 0.20 |
| Baseline non-  coagulation SOFA score | 1.19 | 1.12 – 1.28 | <0.001 | 1.35 | 1.28 – 1.42 | <0.001 |
| VIOLET | Ref | Ref | Ref | Ref | Ref | Ref |
| CLOVERS | 1.20 | 0.75 – 1.94 | 0.44 | 2.10 | 1.42 – 3.09 | <0.001 |
| ASTER | 1.64 | 0.96 – 2.81 | 0.07 | 1.34 | 0.84 – 2.13 | 0.22 |

*Abbreviations:* ARDS, acute respiratory distress syndrome; SOFA, Sequential Organ Failure Assessment; csHR, cause-specific hazard ratio; Ref, reference

Platelet count treated as dichotomous variable using a cut-off of 100,000/μL.

*Versus pulmonary sepsis or no sepsis.

| **Supplemental Table 9. Adjusted cause-specific Cox regression** **model estimates to isolate the contribution of platelet count (treated as continuous variable) to ARDS development and death within 7 days from trial enrollment (competing event) after adjusting for** **sex, age, extrapulmonary sepsis as a primary risk factor for ARDS, hematologic malignancy, baseline non-coagulation SOFA score and trial.** | | | | | | |
| --- | --- | --- | --- | --- | --- | --- |
|  | **ARDS development** | | | **Death** | | |
| **Variable** | **csHR** | **95% Confidence**  **Intervals** | **p value** | **csHR** | **95% Confidence**  **Intervals** | **p value** |
| Platelet count | 1.00 | 0.98 – 1.02 | 0.95 | 0.99 | 0.98 – 1.01 | 0.42 |
| Sex | 1.07 | 0.72 – 1.59 | 0.73 | 1.06 | 0.78 – 1.45 | 0.69 |
| Age | 1.00 | 0.98 – 1.01 | 0.47 | 1.03 | 1.02 – 1.05 | <0.001 |
| Extrapulmonary sepsis* | 0.36 | 0.23 – 0.55 | <0.001 | 0.98 | 0.71 – 1.35 | 0.90 |
| Hematologic malignancy | 0.79 | 0.31 – 1.97 | 0.61 | 1.68 | 0.99 – 2.86 | 0.05 |
| Baseline non-  coagulation SOFA score | 1.20 | 1.12 – 1.28 | <0.001 | 1.35 | 1.29 – 1.43 | <0.001 |
| VIOLET | Ref | Ref | Ref | Ref | Ref | Ref |
| CLOVERS | 1.20 | 0.74 – 1.94 | 0.45 | 2.11 | 1.43 – 3.12 | <0.001 |
| ASTER | 1.65 | 0.96 – 2.82 | 0.07 | 1.39 | 0.87 – 2.21 | 0.17 |

*Abbreviations:* ARDS, acute respiratory distress syndrome; SOFA, Sequential Organ Failure Assessment; csHR, cause-specific hazard ratio; Ref, reference

Platelet count treated as continuous variable using increments of 10,000/μL.

*Versus pulmonary sepsis or no sepsis.

| **Supplemental Table 10. Association of selected platelet count values and development of ARDS after adjusted binary logistic regression analysis, using as reference value a platelet count of 200,000 /μL.** | | | |
| --- | --- | --- | --- |
| **Platelet count (/μL)** | **Odds ratio** | **95% Confidence Intervals** | **p value** |
| 20,000 | 2.244 | 1.073 - 4.692 | 0.032 |
| 50,000 | 1.940 | 1.129 - 3.334 | 0. 016 |
| 100,000 | 1.527 | 1.151 - 2.025 | 0. 003 |
| 150,000 | 1.219 | 1.030 - 1.442 | 0.021 |
| 200,000 | Ref | Ref | Ref |
| 250,000 | 0. 881 | 0. 691 - 1.121 | 0.302 |
| 300,000 | 0. 866 | 0.585 - 1.284 | 0.475 |
| 400,000 | 1.084 | 0. 680 - 1.727 | 0.734 |
| 500,000 | 1.583 | 0. 953 - 2. 629 | 0.076 |
| 600,000 | 2.339 | 1.214 - 4.508 | 0. 011 |
| 700,000 | 3.457 | 1.458 - 8. 195 | 0.005 |
| 800,000 | 5.108 | 1.706 - 15. 289 | 0.004 |
| 900,000 | 7.548 | 1.973 - 28. 878 | 0. 003 |
| 1,000,000 | 11.153 | 2.265 - 54.914 | 0. 003 |

*Abbreviations:* ARDS, acute respiratory distress syndrome; Ref, reference

The model included the following covariates: sex, age, extrapulmonary sepsis as a primary risk factor for ARDS, hematologic malignancy, baseline non-coagulation Sequential Organ Failure Assessment (SOFA) score and trial.

**Supplemental Figure 1. Histograms with rug strikes depicting distribution of platelet count values among participants in the three randomized controlled trials (VIOLET, CLOVERS and ASTER) of the secondary analysis.**

**
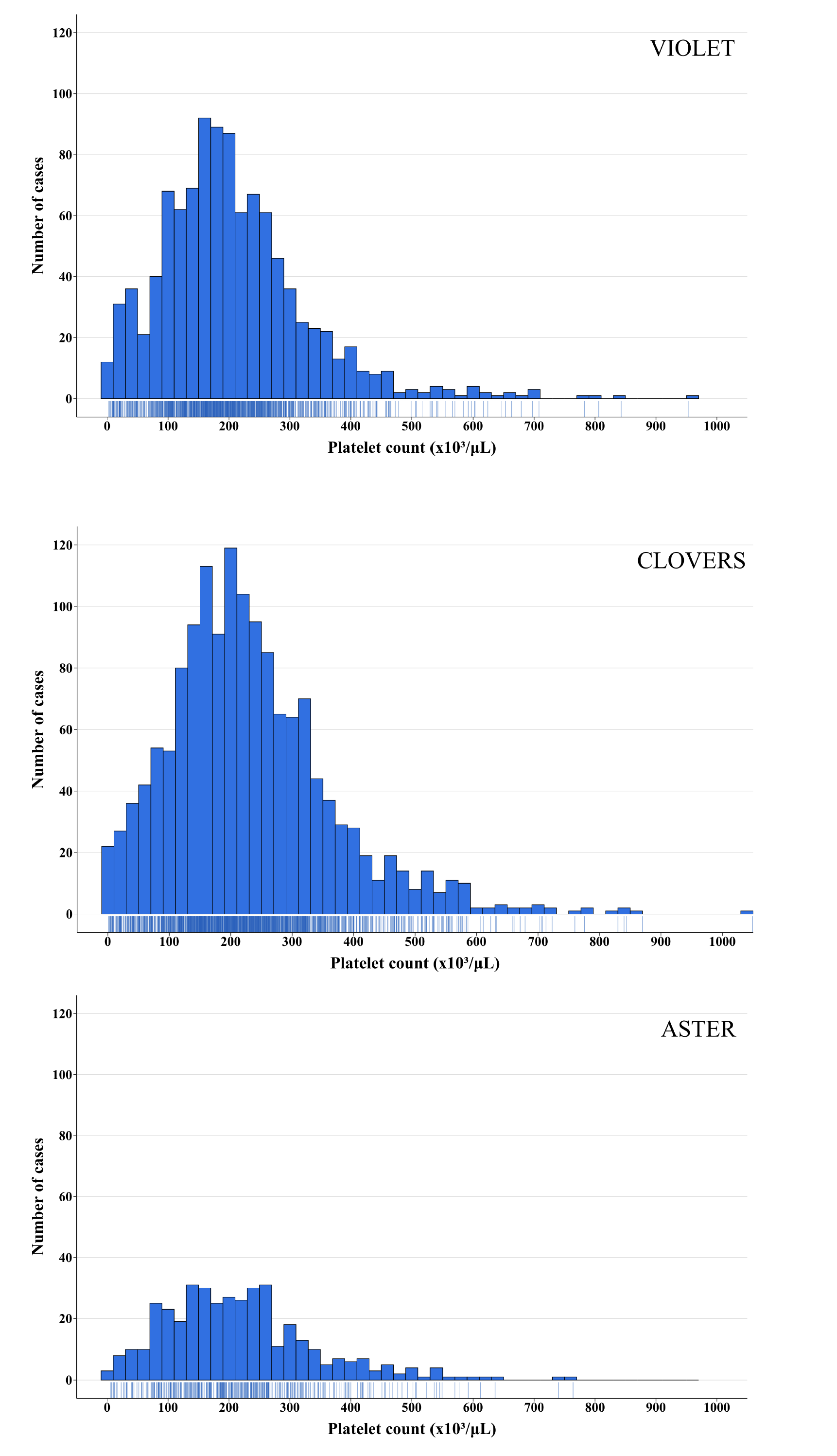
**

**Supplemental Figure 2. Plots of Martingale residuals of the adjusted cause-specific Cox regression model, which considered platelet count as a dichotomous variable against continuous covariates; namely, age and baseline non-coagulation Sequential Organ Failure Assessment score.**


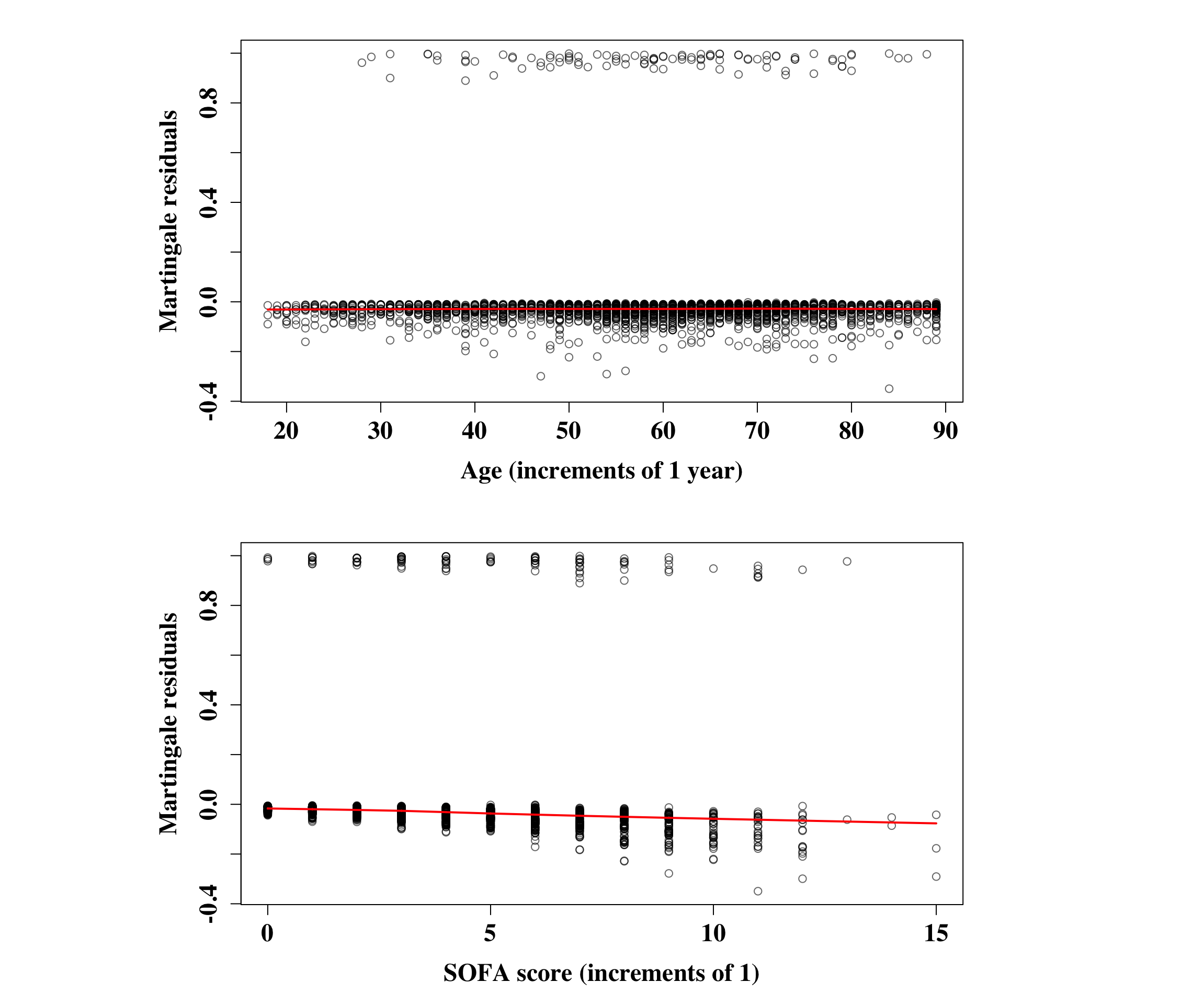


**Supplemental Figure 3. Plots of Martingale residuals of the adjusted cause-specific Cox regression model, which considered platelet count as a continuous variable against continuous covariates; namely, platelet count, age and baseline non-coagulation** **Sequential Organ Failure Assessment score.**


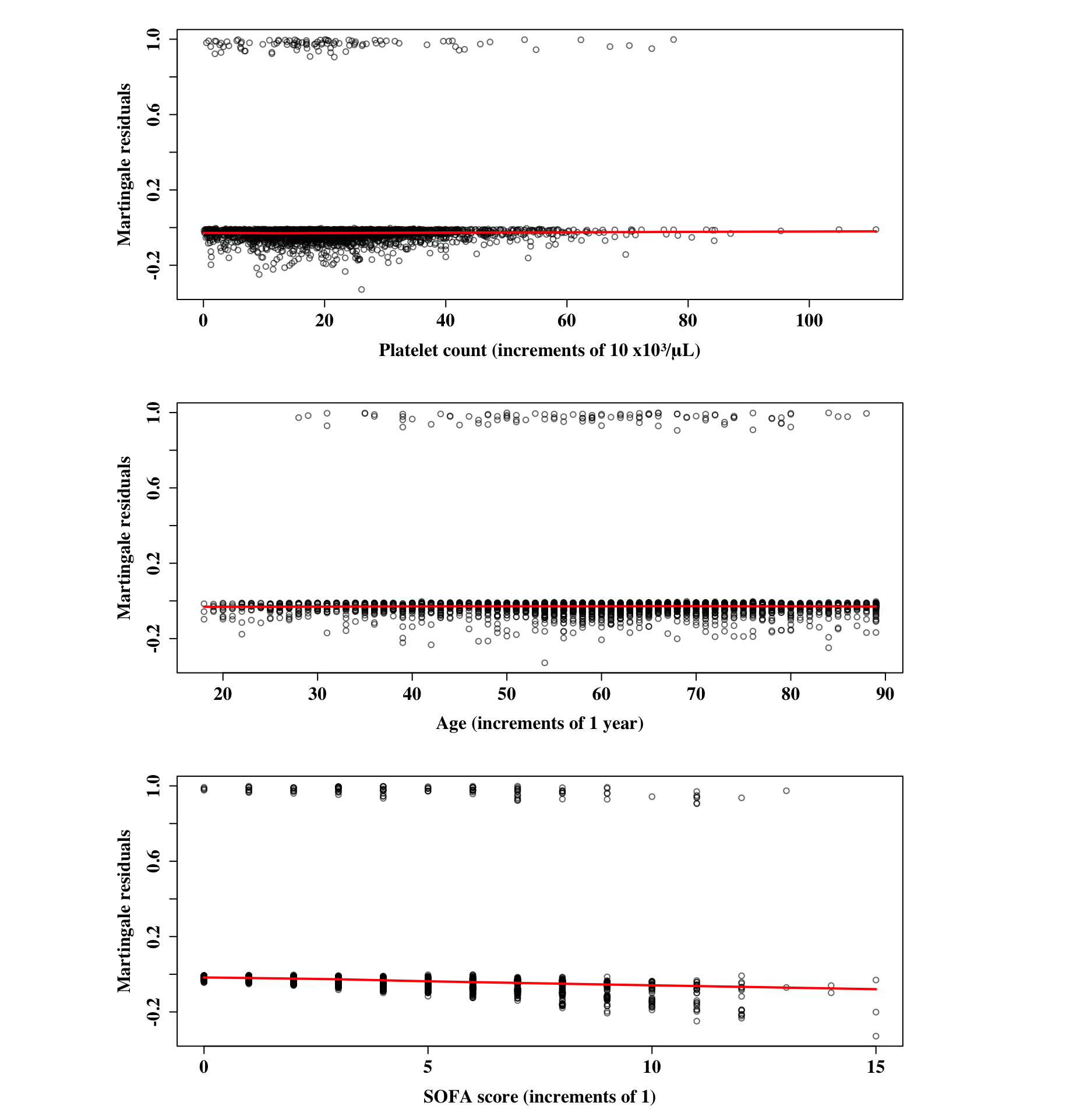


**Supplemental Figure 4. Scatter plots of the Schoenfeld residuals for the independent variables included in the adjusted cause-specific Cox regression model, which considered platelet count as a dichotomous variable, against follow-up time.**


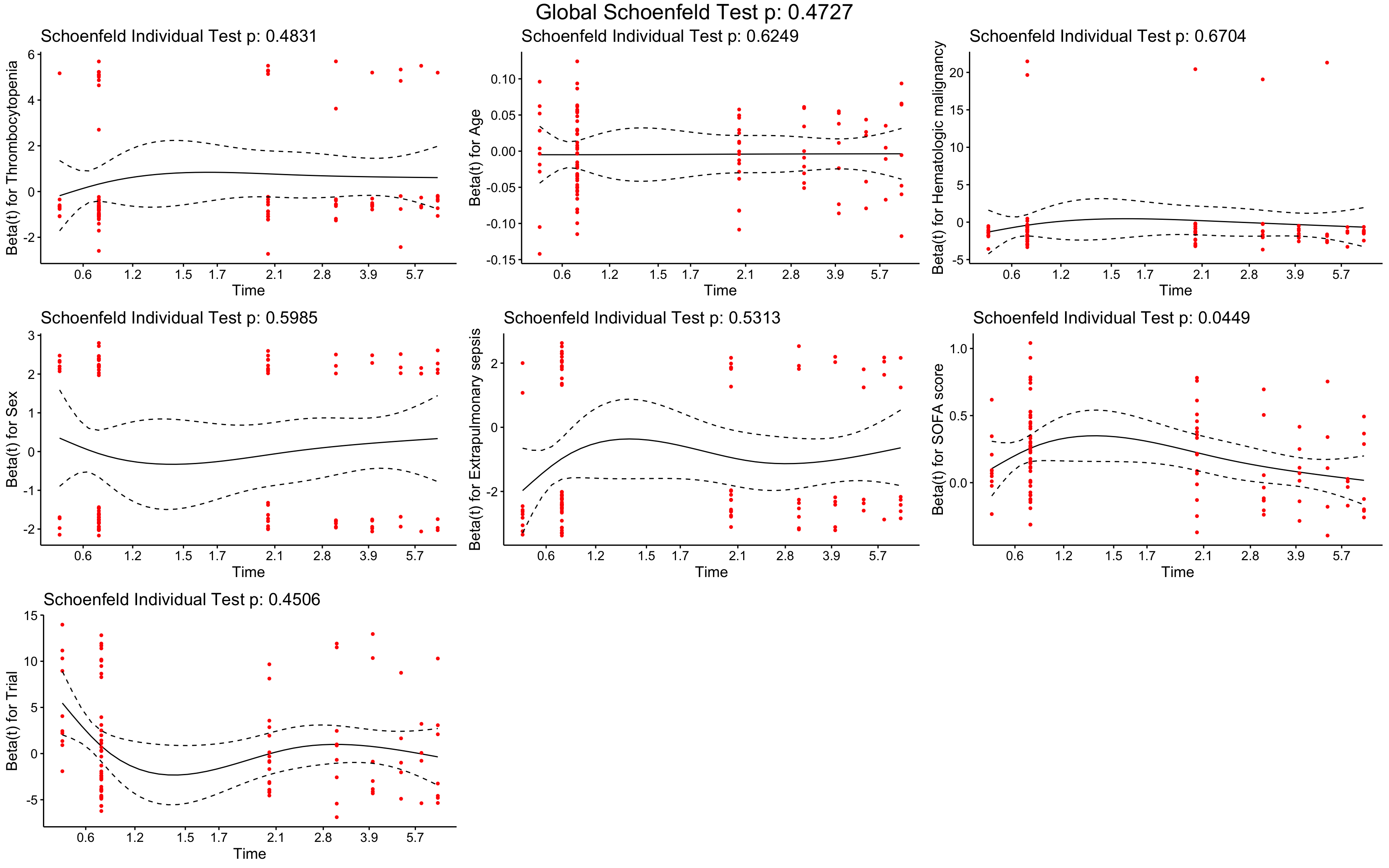


**Supplemental Figure 5. Scatter plots of the Schoenfeld residuals for the independent variables included in the adjusted cause-specific Cox regression model, which considered platelet count as a continuous variable, against follow-up time.**


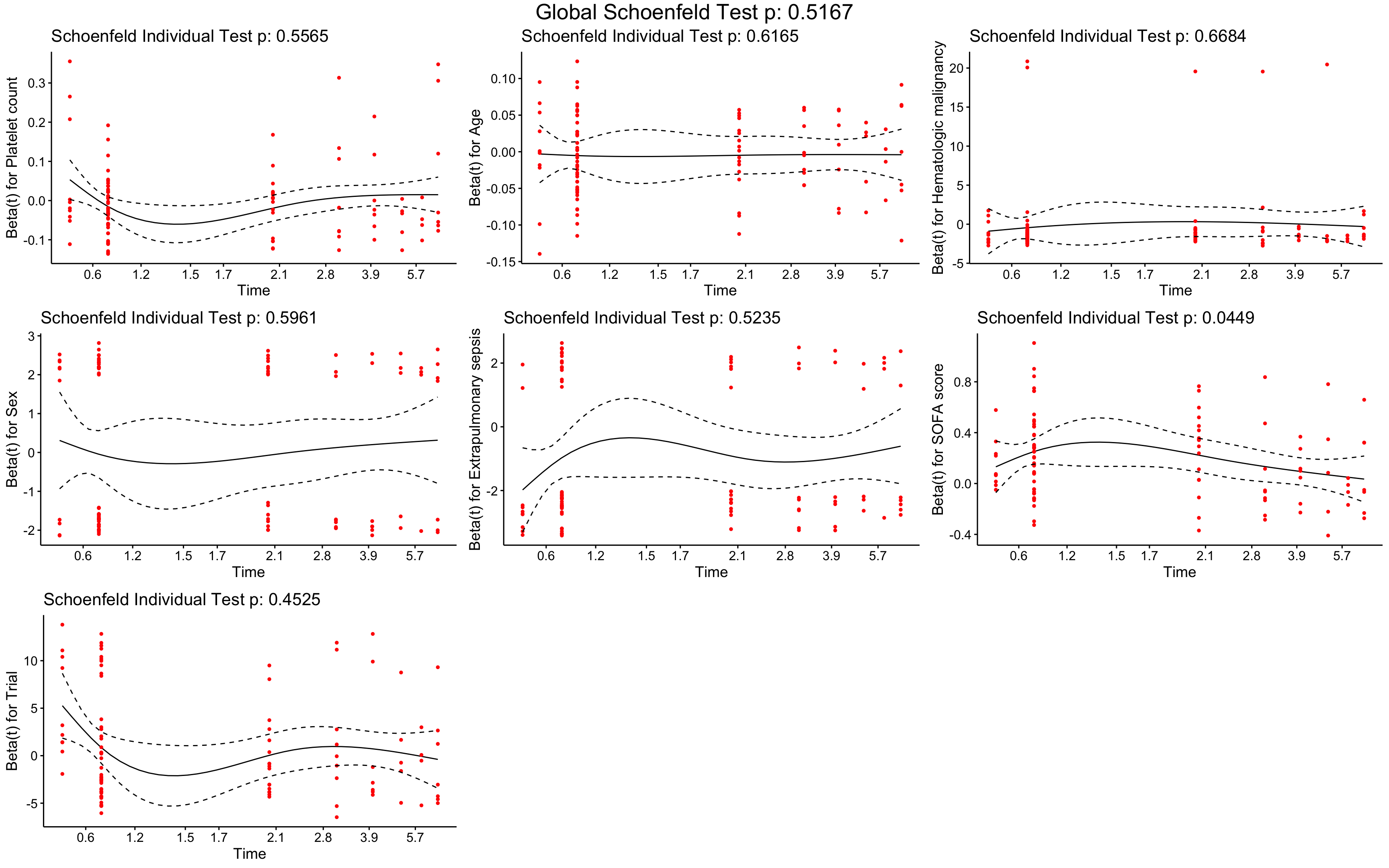


**Supplemental Figure 6. Adjusted cause-specific cumulative incidence curves comparing the risk of ARDS development between patients with versus without thrombocytopenia (defined as <100,000 platelets/μL).** *Abbreviation*: ARDS, acute respiratory distress syndrome. The model included the following covariates: sex, age, extrapulmonary sepsis as a primary risk factor for ARDS, hematologic malignancy, baseline non-coagulation Sequential Organ Failure Assessment (SOFA) score and trial.

**
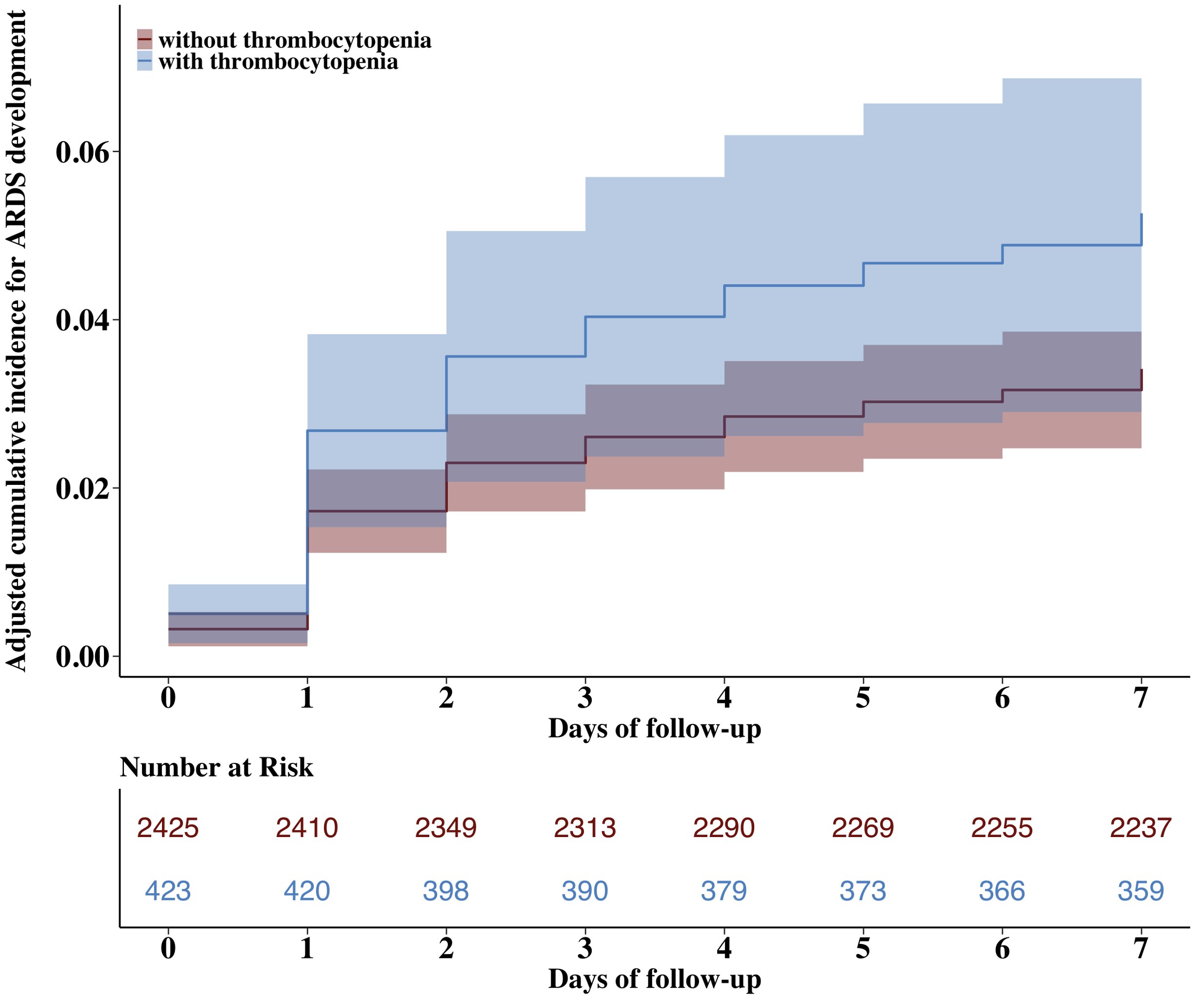
**

**Supplemental Figure 7. Modeled odds ratio for ARDS development according to platelet count after adjusted binary logistic regression analysis.** *Abbreviation:* ARDS, acute respiratory distress syndrome. The model included the following covariates: sex, age, extrapulmonary sepsis as a primary risk factor for ARDS, hematologic malignancy, baseline non-coagulation Sequential Organ Failure Assessment (SOFA) score and trial. As reference value we considered a platelet count of 200,000/μL. The gray areas represent the 95% confidence intervals.


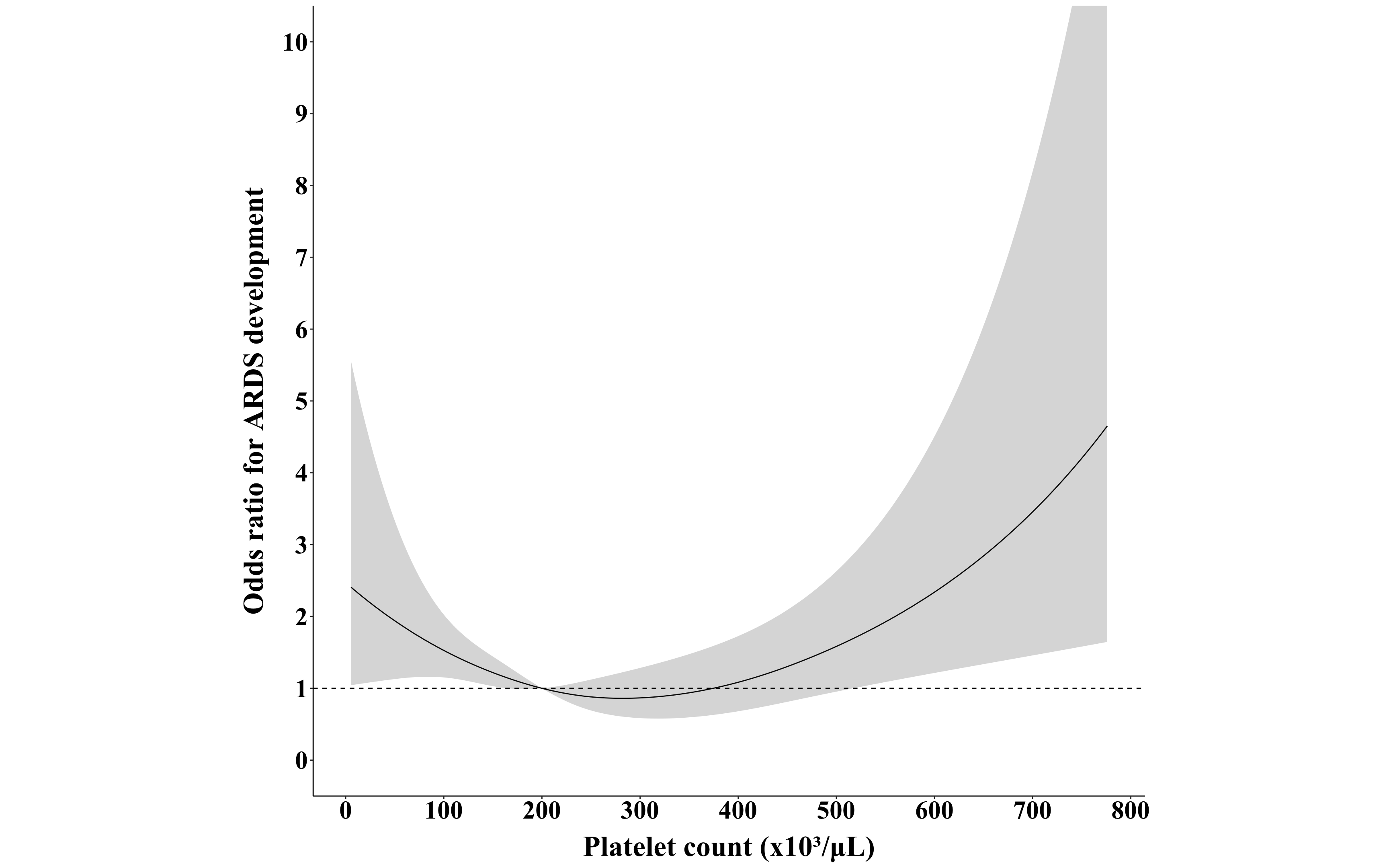

Supplement: Supplementary file 1 — Supplementary Material 1. [file 12931_2025_3444_MOESM1_ESM.docx]
